# Allergen-Induced Dermatitis Causes Alterations in Cutaneous Retinoid-Mediated Signaling in Mice

**DOI:** 10.1371/journal.pone.0071244

**Published:** 2013-08-15

**Authors:** Janine Gericke, Jan Ittensohn, Johanna Mihály, Sandrine Dubrac, Ralph Rühl

**Affiliations:** 1 Laboratory of Nutritional Bioactivation and Bioanalysis, Department of Biochemistry and Molecular Biology, University of Debrecen, Debrecen, Hungary; 2 Paprika Bioanalytics BT, Debrecen, Hungary; 3 Epidermal Biology Laboratory, Department of Dermatology, Innsbruck Medical University, Innsbruck, Austria; 4 Apoptosis and Genomics Research Group of the Hungarian Academy of Science, Debrecen, Hungary; Laboratoire de Biologie du Développement de Villefranche-sur-Mer, France

## Abstract

Nuclear receptor-mediated signaling via RARs and PPARδ is involved in the regulation of skin homeostasis. Moreover, activation of both RAR and PPARδ was shown to alter skin inflammation. Endogenous all-*trans* retinoic acid (ATRA) can activate both receptors depending on specific transport proteins: Fabp5 initiates PPARδ signaling whereas Crabp2 promotes RAR signaling. Repetitive topical applications of ovalbumin (OVA) in combination with intraperitoneal injections of OVA or only intraperitoneal OVA applications were used to induce allergic dermatitis. In our mouse model, expression of IL-4, and Hbegf increased whereas expression of involucrin, Abca12 and Spink5 decreased in inflamed skin, demonstrating altered immune response and epidermal barrier homeostasis. Comprehensive gene expression analysis showed alterations of the cutaneous retinoid metabolism and retinoid-mediated signaling in allergic skin immune response. Notably, ATRA synthesis was increased as indicated by the elevated expression of retinaldehyde dehydrogenases and increased levels of ATRA. Consequently, the expression pattern of genes downstream to RAR was altered. Furthermore, the increased ratio of Fabp5 vs. Crabp2 may indicate an up-regulation of the PPARδ pathway in allergen-induced dermatitis in addition to the altered RAR signaling. Thus, our findings suggest that ATRA levels, RAR-mediated signaling and signaling involved in PPARδ pathways are mainly increased in allergen-induced dermatitis and may contribute to the development and/or maintenance of allergic skin diseases.

## Introduction

Atopic dermatitis (AD) is the most common inflammatory skin condition, predominantly affecting infants and children and characterized by pruritus, eczematous lesions, and skin dryness. Furthermore, the disease is commonly associated with allergic conditions such as allergic rhinitis and asthma. AD affects 10–30% of children and 2–10% of adults in industrialized countries, with a marked increase in AD prevalence during the past 30 years [1]–[3]. While various studies reported an “outside-inside-outside” pathogenic mechanism of AD [4]–[6], its exact pathogenesis is not yet fully elucidated.

Vitamin A and its derivatives, the retinoids, are essential for skin physiology [7] through their role in the regulation of several aspects of skin cell proliferation, differentiation, apoptosis, immune regulation and epidermal barrier function [8], [9]. Noticeably, alterations of retinoid metabolism and signaling were found in skin of patients with various skin diseases, such as psoriasis [10], ichthyosis [11], and recently by our group in AD [12]. Thereby, it is unclear whether these alterations are the trigger or if they are consequence of these skin diseases. Furthermore, it was previously shown that retinoids are able to modify the immune phenotype of atopic diseases such as AD [13], [14].

Retinoids mediate their function mainly via signaling through nuclear hormone receptors, i.e. retinoic acid receptor (RAR) α, β, and γ and retinoid X receptor (RXR) α, β, and γ. RARs and other nuclear receptors, like peroxisome proliferator-activated receptors (PPAR) α, δ (β), and γ, function as ligand-dependent transcription factors and regulate the expression of various genes after heterodimerization with RXR [15]. Within these three receptor families, RARγ, RXRα and PPARδ are the most abundant subtypes present in skin [16], [17].

PPARmediated pathways are important in skin physiology because they are involved in epidermal barrier recovery, keratinocyte differentiation and lipid synthesis [16]. For example, overexpression of PPARδ in the epidermis causes a psoriasis-like skin disease featuring hyperproliferation of keratinocytes, dendritic cell accumulation, and endothelial activation [18]. Interestingly, a cross-talk exists between RAR and PPARδ pathways. Indeed, RARγ and PPARδ can both be activated by the endogenous RAR ligand all-*trans* retinoic acid (ATRA), depending on specific transport proteins. The cellular retinoic acid binding protein 2 (Crabp2) initiates RAR signaling, whereas the fatty acid-binding protein 5 (Fabp5) promotes PPARδ-mediated signaling after ATRA-binding [19], [20]. However, these findings are controversially discussed in the literature [21]–[23]. Moreover, PPARδ activation has been reported at high ATRA concentrations suggesting that tissue levels of ATRA can determine which nuclear receptor pathways are up-regulated and thereby influence the gene expression profile [19], [20].

The aim of the present study was to determine whether the induction of allergic immune responses in the skin by combined systemic and topical treatments with ovalbumin (OVA) is able to modify retinoid metabolism and retinoid-mediated signaling in the skin of mice. Furthermore, we studied the effects of systemic OVA sensitization without further topical sensitization on skin retinoid metabolism as a potential model for an inside-outside patho-mechanism of allergic skin disorders. Our final aim was to determine via which nuclear hormone receptor-mediated pathways retinoid signaling might be regulated to modify skin inflammation and homeostasis in allergen-induced dermatitis.

## Materials and Methods

### Sensitization of Mice

8–10 weeks old female BALB/c mice, were obtained from and housed within the animal facility of the University of Debrecen, Hungary. Animals were maintained in single cages with standard animal chow and water *ad libitum*. All experimental procedures were approved by the Committee of Animal Research of the University of Debrecen, Hungary (Approval number: 25/2006 DEMÁB).

**Figure 1:**
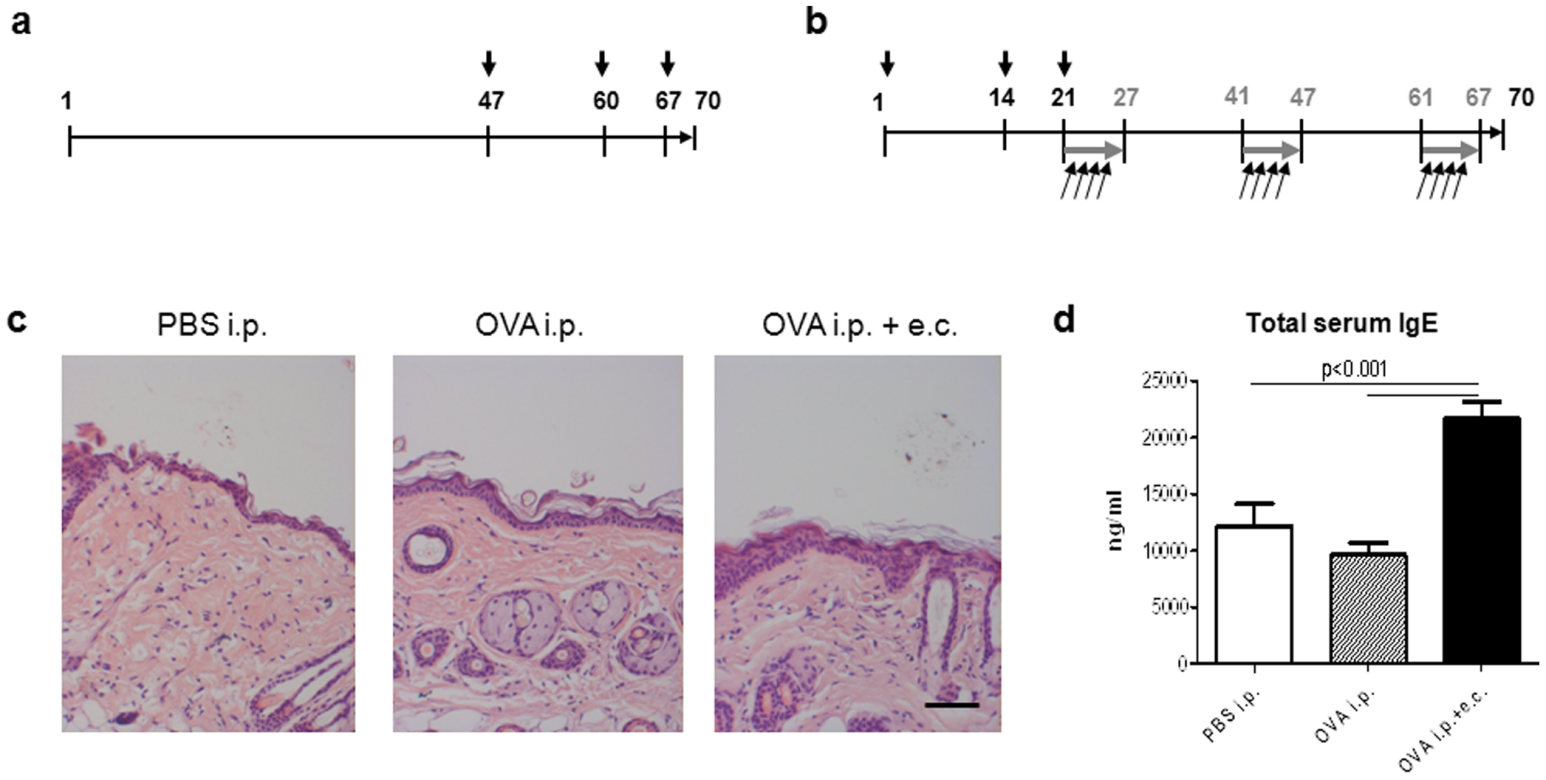
Sensitization protocol, histological analysis, and IgE serum levels. (**a**) Mice were sensitized i.p. with 10 µg OVA adsorbed to 1.5 mg Al(OH)_3_ or with phosphate-buffered saline (PBS; control) on days 47, 60 and 67 (black arrows). (**b**) A third group of mice was sensitized i.p. with 10 µg OVA adsorbed to 1.5 mg Al(OH)_3_ on days 1, 14 and 21 (black arrows) followed by e.c. OVA exposure for three 1-week periods (grey arrows) separated by 2-weeks intervals. Each mouse received a weekly e.c. dose of 100 µg OVA adsorbed to 1.5 mg Al(OH)_3_ in 100 µl PBS on shaved back skin divided into four applications of 25 µl every other day of one week (black angular arrows). Three days after the last treatment (day 70), mice were sacrificed and skin and serum samples were collected. (**c**) Hematoxylin and Eosin staining of five-micrometer skin sections obtained from treated dorsal skin sites. Images were taken at ×10 magnification (scale bar = 50 µm). (**d**) Total IgE levels were determined in the serum of mice treated systemically with or without additional topical sensitization with OVA. Data are presented as mean values ± SEM of three independent measurements with triplicate determination of n = 8 mice/group. Statistical significance (*p*) is based on one-way ANOVA followed by Tukey’s multiple comparison test.

Sensitization of mice was performed by repetitive systemic application of OVA and allergen-induced dermatitis based on a model previously reported [24], [25]. Briefly, mice were sensitized at days 47, 60 and 67 with 10 µg OVA intraperitoneally (i.p.) (Sigma-Aldrich, Budapest, Hungary) adsorbed to 1.5 mg aluminum hydroxide (Al(OH)_3_) (Thermoscientific, Budapest, H) or with phosphate-buffered saline (PBS; control) (Figure 1a). For combined treatment [24], mice were sensitized i.p. on days 1, 14 and 21 with 10 µg OVA adsorbed to 1.5 mg Al(OH)_3_. This was followed by topical application of 100 µg OVA adsorbed to 1.5 mg Al(OH)_3_ in 100 µl PBS (weekly dose) onto shaved back skin, divided into four applications of 25 µl every other day of one week (Figure 1b). Epicutaneous (e.c.) treatment was repeated for a total exposure of three weeks separated by two-week intervals. Three days after the last treatment (day 70) mice were euthanized: skin and serum samples were collected and kept at −80°C until analyses.

### Histological Analysis

Skin biopsies of were taken from similar body sites, fixed overnight with 4% paraformaldehyde at 4°C, and embedded in paraffin. Five-micrometer sections were stained with hematoxylin and eosin (H&E) or Giemsa. Mast cells were quantified under a light microscope using 20x lenses and a calibrated grid (six fields per sample) while epidermal thickness was measured using 10x lenses (five measurements per mouse).

### Immunohistochemical Analysis

To quantitate lymphocytes and dendritic cells in the skin, CD3^+^, CD4^+^, CD8^+^, MHC-class II^+^ and CD11c^+^ cells in frozen skin sections were counted under an Olympus BX60 epifluorescence microscope using 40× objective lenses and a calibrated grid (six fields per section). For detailed information on used antibodies and staining procedure refer to Materials and Methods S1a. After fixation in paraformaldehyde, five-micrometer sections were incubated with a rabbit anti-mouse Fabp5 polyclonal antibody (1∶50; ProteinTech, Chicago, IL) following manufacturer’s directions after antigen retrieval (Materials and Methods S1b).

### Total IgE Levels in Serum

Sera were collected at day 70 of the experiment (Figure 1a and b) and kept at −80°C until analysis. Plasma IgE concentration was measured using the mouse ELISA kit from BD-Pharmingen.

### RNA Preparation and Reverse Transcription

Total RNA was isolated from frozen skin using Tri® reagent (Molecular Research Center Inc., Cincinnati, OH) following the manufacturer’s instructions. 750 ng of total RNA were reverse transcribed into cDNA in a 30 µl reaction using the High Capacity cDNA Reverse Transcription Kit (Life Technologies, Budapest, H) according to the manufacturer’s protocol.

### Analysis of mRNA Expression

mRNA expression in skin was determined by means of quantitative real time-PCR (qRT-PCR) and TaqMan® Low Density Arrays (TLDA) on an ABI Prism 7900. qRT-PCR measurements were performed in triplicate using pre-designed TaqMan® Gene Expression Assays and reagents; TaqMan® Low Density Array cards were used for duplicate determinations using TaqMan® Gene Expression Master Mix (all Applied Biosystems Applera Hungary, Budapest, H). Relative quantification of mRNA expression was achieved using the comparative C_T_ method and values were normalized to cyclophilin A mRNA. Gene expression values below detection limit were assumed to be zero for the purpose of statistical analysis.

### Determination of FABP5 Protein in Skin

FABP5 protein levels were determined in protein lysates prepared from whole mouse skin according to the protocol indicated in Materials and Methods S2 using the rabbit FABP5 polyclonal antibody purchased from ProteinTech (Chicago, IL).

### Determination of Retinol and ATRA Levels in Skin

Concentrations of ATRA and retinol were determined in mouse skin samples by our high performance liquid chromatography mass spectrometry - mass spectrometry (HPLC MS-MS) method as described in Materials and Methods S3 [26].

### Cytokine Levels in Serum

Levels of TSLP, IL-4 and IL-12 (p70) were determined in serum using Quantikine Mouse Immunoassays (R&D Systems, Abingdon, UK), according to the manufacturer’s protocol. Assay sensitivity was 2.63 pg/mL for TSLP, <2 pg/mL for IL-4, and <2.5 pg/mL for IL-12.

### Statistical Analysis

Data are indicated as mean ± SEM. Statistical analysis was performed using one-way ANOVA followed by Tukey correction. Significance of HPLC MS-MS results was determined using Student’s *t*-test. Differences were considered significant at *p*<0.05.

## Results

### Systemic Sensitization with OVA Induces Mild Allergen-induced Dermatitis When Compared to Additional Topical OVA Applications

BALB/c mice were systemically sensitized with OVA in addition or not to topical sensitization onto shaved back skin (Figure 1a and b) and compared with PBS-injected mice (controls). Sensitization with OVA induced mild but statistically significant focal hyperplasia with a two-fold (OVA i.p.; *p*<0.001 vs. PBS i.p.) or three-fold (OVA i.p.+e.c.; *p*<0.001 vs. PBS i.p.; *p*<0.001 vs. OVA i.p.) increase in epidermal thickness, respectively (Figure 1c). Histological analysis further revealed scaly skin in both OVA-sensitized groups (Figure 1c). Interestingly, repeated systemic sensitization with OVA did not alter total serum IgE levels compared to control mice (Figure 1d). However, combined systemic and topical sensitization leading to allergen-induced dermatitis resulted in significantly increased total serum IgE (21.696±1.655 ng/ml) when compared to controls and systemically sensitized mice (Figure 1d). Furthermore, inflammatory cells infiltrating the skin were characterized by Giemsa staining and immunohistochemical methods. Numbers of mast cells were significantly elevated in the dermis of both OVA-sensitized groups, while the increase in macrophages, dermal dendritic cells and CD4^+^ lymphocytes reached statistical significance only in allergen-induced dermatitis (Table 1). Sensitization with OVA induced elevated numbers of CD3^+^ lymphocytes in both, the dermis and epidermis (data not shown). Only few CD8^+^ lymphocytes could be detected in skin sections of OVA-sensitized mice (data not shown). Eosinophils could only be found in the dermis of OVA-sensitized mice and predominantly in allergen-induced dermatitis (Table 1). In the epidermis, several Langerhans cells exhibiting activated morphology could be observed in OVA-sensitized mice (data not shown). Altogether, numbers of mast cells, macrophages and dermal dendritic cells were significantly higher only in allergen-induced dermatitis (Table 1). Thus, repeated systemic sensitization with OVA is sufficient to initiate an inflammatory immune response in the skin whereas additional topical sensitization is required to induce full allergen-mediated dermatitis in mice [24], [25].

**Table 1:** Systemic sensitization with ovalbumin (OVA) induced mild allergic dermatitis when compared to additional topical OVA applications.

	Sensitization		
Analysis	PBS i.p.	OVA i.p.	OVA i.p.+e.c.
*Inflammatory cell infiltrate in skin* 1			
Mast cells^2^	7±1	27±2***	45±3*** ^###^
Eosinophils^2^	0±0	5±1*	9±2***
Macrophages (MHC II^+^CD11c^−^)^3^	307±16	369±21	474±14*** ##
Dermal dendritic cells (MHC II^+^CD11c^+^)^3^	40±3	59±4	90±10*** #
CD3^+^ lymphocytes^3^	164±13	240±15**	285±10***
CD4^+^ lymphocytes^3^	4±1	11±2	14±2**
*Cytokine serum levels (pg/mL)* 4			
Th1-type			
Interleukin 12 p70 (IL-12)	7.4±3.9	6.5±4	8.4±5.6
Th2-type			
Interleukin 4 (IL-4)	73±22	179±24*	198±15**
Thymic stromal lymphopoietin (Tslp)	3.5±0.5	3.7±1.2	3.4±0.4

e.c., epicutaneous; i.p., intraperitoneal; OVA, ovalbumin; PBS, phosphate-buffered saline; Th, T-helper cell.

Data are indicated as mean ± SEM. Statistical significance (*p*) was tested using one-way ANOVA followed by Tukey’s multiple comparison test.

* *p*<0.05,

** *p*<0.01,

*** *p*<0.001, versus PBS i.p.;

# *p*<0.05,

## *p*<0.01, and ^###^ *p*<0.001, versus OVA i.p.

1 Positively stained cells were counted in six fields per section of n = 5 (IHC-staining) or n = 8 animals (Giemsa-staining), respectively, and expressed as cells per mm^2^.

2 Determined in Giemsa-stained skin sections.

3 Determined in IHC-stained skin sections.

4 Cytokine levels in sera were determined as triplicate measurements of n = 4 mice per group using Quantikine mouse immunoassay kits. Data are indicated as pg/ml.

### Expression of Th1- and Th2-type Immune Response Genes is only Altered in Allergen-induced Dermatitis

Expression of genes relevant for Th1- or Th2-type immune response was analyzed in the skin of mice by qRT-PCR and TLDA. As shown in Table 2, gene expression levels of the inflammation-associated keratin 17 (Krt17) was moderately increased in the skin of OVA-sensitized mice. In contrast, expression of Th1 related genes including interferon γ (Ifng) and interleukin 12A (Il12a) and of Th2 associated genes including interleukin 4 (Il4), interleukin 10 (Il10), chemokine ligand 11 (eotaxin 1; Ccl11) and its corresponding chemokine receptor 3 (Ccr3) was significantly elevated only in the skin of mice with allergen-induced dermatitis (Table 2). Altogether, these results (Tables 1 and 2) show that only combined systemic and topical sensitizations with OVA fully recapitulate allergen-induced dermatitis symptoms, which are also found in chronic AD [27]–[31].

**Table 2:** Systemic and topical OVA sensitization results in inflammation, disturbs epidermal barrier homeostasis, and induces PPARδ target gene expression in skin.

		Fold change	
Gene name	Symbol	OVA i.p.	OVA i.p.+e.c.
**Immune response**			
Keratin 17	Krt17	1.6±0.2*	1.7±0.2**
*Th1-type*			
Interleukin 12A	Il12a	1639±719	2613±740*
Interferon γ	Ifng	11.1±5.4	22.1±2.8**
*Th2-type*			
Interleukin 4	Il4	11.7±4.5	92.7±30.9** #
Interleukin 10	Il10	12.4±5.5	32.7±13.1*
Chemokine (CC motif) ligand 11/eotaxin 1	Ccl11	0.2±0.2	13.8±5.1* #
Chemokine (CC motif) receptor 3	Ccr3	63.7±34.4	1763±534*** ##
**Epidermal barrier homeostasis**			
ATP-binding cassette A12^1^	Abca12	0.3±0***	0.6±0.1*** ##
Involucrin	Ivl	0.6±0.1*	0.4±0***
Loricrin	Lor	0.5±0*	0.8±0.1#
Serine peptidase inhibitor, Kazal-type 5	Spink5	0.2±0***	0.4±0***
Matrix metalloproteinase 9	Mmp9	0.1±0***	0.6±0.1##
S100 calcium binding protein A7A/psoriasin	S100a7a	2.6±0.3**	2.1±0.3*
3-Hydroxy-3-methylglutaryl-CoA synthase 2^1^	Hmgcs2	0.4±0.1	4.4±1.1** ##
Serine palmitoyltransferase long chain base subunit 2	Sptlc2	1.2±0.3	33.8±10.6** ##
UDP-glucose ceramide glucosyltransferase	Ugcg	196±16.9***	161±14.4***
Alkaline ceramidase 1	Acer1	2.2±0.6	3.9±0.3*** #
**PPARδ signaling**			
Peroxisome proliferator-activated receptor δ	Ppard	0.4±0**	0.6±0*
Fatty acid-binding protein 5	Fabp5	1.7±0.3	2.2±0.2**
ATP-binding cassette A12^1^	Abca12	0.3±0***	0.6±0.1*** ##
Keratin 6B	Krt6b	5.8±0.6	632±177** ##
Keratin 16	Krt16	0.5±0	5.7±1.2*** ^###^
Heparin-binding EGF-like growth factor	Hbegf	1.1±0.1	2.1±0.3* #
3-Hydroxy-3-methylglutaryl-CoA synthase 2^1^	Hmgcs2	0.4±0.1	4.4±1.1** ##

e.c., epicutaneous; i.p., intraperitoneal; OVA, ovalbumin; Th, T-helper cell.

1 Enumerated twice because the gene is relevant for two pathways.

Fold change data are expressed as mean ± SEM (n = 6) and were determined in skin specimen of sensitized mice by TLDA or qRT-PCR. Statistical significance (*p*) was tested using one-way ANOVA followed by Tukey’s multiple comparison test.

* *p*<0.05,

** *p*<0.01,

*** *p*<0.001, versus control (PBS i.p.);

# *p*<0.05,

## *p*<0.01, and ^###^ *p*<0.001, versus OVA i.p.

### Systemic Sensitization with OVA is Sufficient to Modify Expression of Genes Involved in Epidermal Barrier Homeostasis

#### Decreased expression of genes related to the epidermal protein compartment

mRNA levels of several genes involved in epidermal barrier formation and maintenance, such as keratinocyte differentiation markers involucrin (Ivl) and loricrin (Lor), matrix metalloproteinase 9 (Mmp9) and serine peptidase inhibitor Kazal-type 5 (Spink5) were significantly decreased in the skin of OVAtreated mice (Table 2). Notably, expression levels of all tested genes, except for psoriasin, were lower in the OVA-treated mice (Table 2). Indeed, psoriasin (S100a7a) expression was induced in mice treated with OVA (Table 2).

#### Increased expression of genes related to the epidermal lipid compartment

In contrast to the protein compartment, genes related to the epidermal lipid compartment were mainly up-regulated in mouse skin after OVA challenges. In fact, mRNA expression levels of 3-Hydroxy-3-methylglutaryl-CoA synthase 2 (Hmgcs2), involved in cholesterol synthesis, of serine palmitoyltransferase 2 (Sptlc2), UDP-glucose ceramide glucosyltransferase (Ugcg), both catalyzing the synthesis of ceramides and glycosyl-ceramides, and of alkaline ceramidase 1 (Acer1), which is responsible for ceramide degradation, were significantly elevated in the skin of mice with allergen-induced dermatitis and/or solely systemic OVA sensitization (Table 2). Noticeably, only expression of ATP-binding cassette A12 (Abca12), which is responsible for lipid loading into lamellar bodies, was decreased in OVA-treated groups (Table 2). Taken together, our results suggest that systemic sensitization with OVA is sufficient to decrease expression of differentiation markers and to increase expression of proteins involved in the cutaneous lipid metabolism. This, in turn, might lead to an impaired epidermal barrier function.

### Systemic Sensitization with OVA Triggers IL-4 Serum Levels

**Figure 2:**
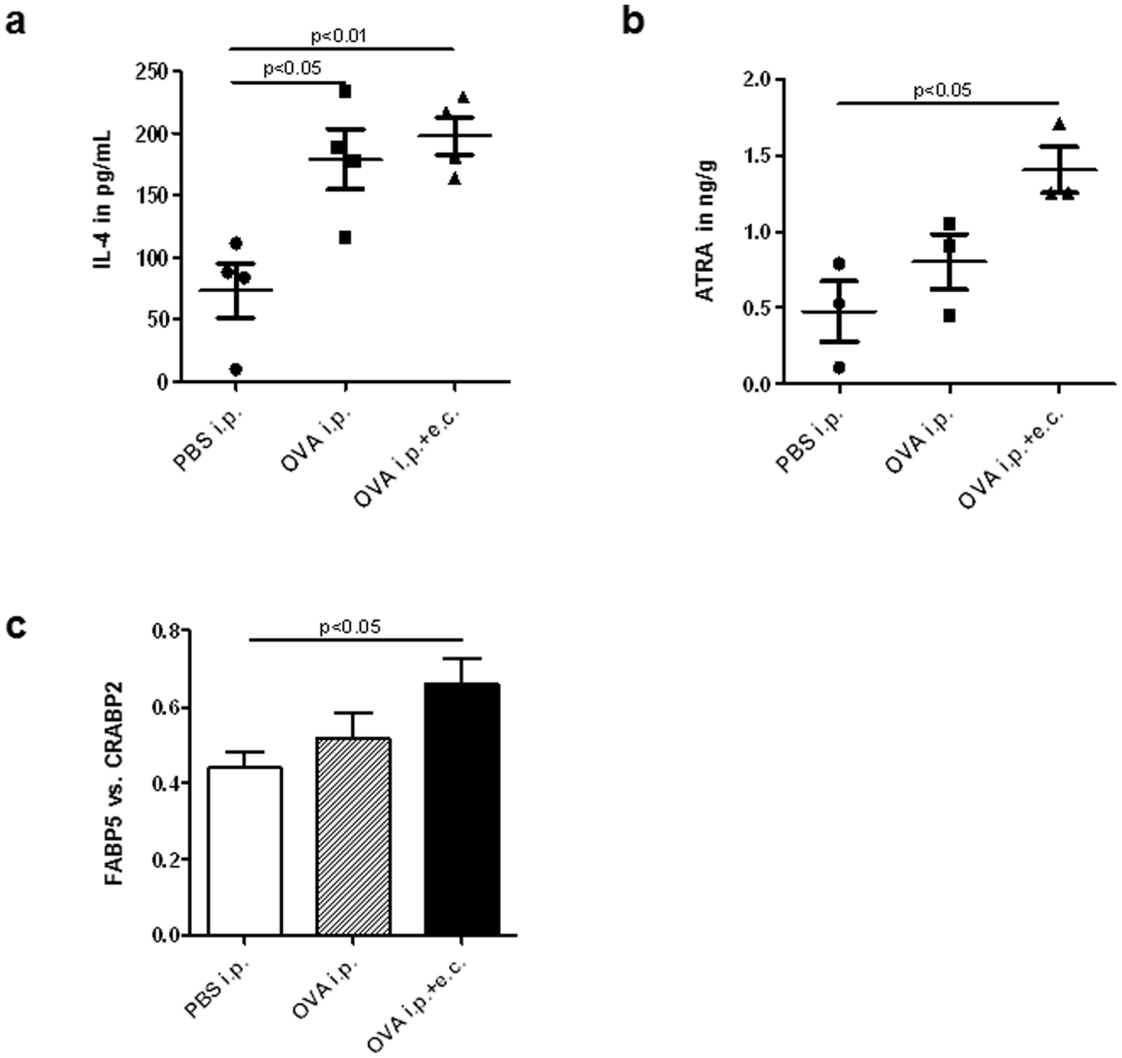
Serum levels of IL-4 and ATRA and the Fabp5 vs. Crabp2 ratio are increased in skin after OVA sensitizations. (**a**) IL-4 serum levels after systemic with or without additional topical OVA sensitization (n = 8). (**b**) ATRA levels in mouse skin determined by HPLC MS-MS method upon systemic (i.p.) and systemic plus topical (i.p.+e.c.) OVA sensitization (n = 3/group). (**c**) Ratio of Fabp5 vs. Crabp2 expression in the skin of OVA-treated mice (n = 6/group) compared to control mice (PBS i.p.). Data are presented as mean values ± SEM. Statistical significance (*p*) is based on one-way ANOVA followed by Tukey’s multiple comparison test for gene expression results and ELISA data. For HPLC MS-MS results, significance was determined using Student’s *t*-test.

Levels of the cytokines IL-4, TSLP and IL-12 (IL-12p70) were determined in the sera of OVA-sensitized and control mice. IL-4 but not TSLP was significantly increased in both OVA-treated groups (Table 1, Figure 2a). Serum levels of IL-12, a Th1-type cytokine, remained similar in all groups (Table 1).

### Retinoic Acid Levels and Retinoid Metabolism are Increased in Allergen-induced Dermatitis

While retinoid-mediated signaling in skin is involved in several physiological processes, little is known about RAR-mediated signaling in inflammatory skin diseases [12]. By means of our mouse model we aimed to investigate whether repeated systemic and combined systemic and topical sensitizations with OVA are able to induce changes in retinoid metabolism and retinoid-mediated signaling on the gene expression level in skin. After sensitization, expression of short chain dehydrogenase/reductase 16C5 (Sdr16c5), responsible for the oxidation of retinol to retinal, was induced compared to controls while expression of retinol dehydrogenase 10 (Rdh10) remained unchanged (Table 3). In contrast, expression of enzymes responsible for the conversion of retinal to the bioactive vitamin A derivative ATRA (aldehyde dehydrogenases; Aldh1a1, 1a2, and 1a3) was significantly increased only in allergen-induced dermatitis (Table 3). Importantly, we found significantly elevated concentrations of ATRA only in the skin of mice with allergen-induced dermatitis (Figure 2b, Table S1), while retinol levels remained unchanged (Table S1). Different retinoid receptors mediate the effects of ATRA in skin. Parallel to ATRA skin content, elevated mRNA levels of RARγ and RXRα, both the most abundant retinoid receptors in skin, were evidenced only in allergen-induced dermatitis (Table 3). To further investigate the induction of retinoid-mediated signaling in sensitized skin, we next assessed expression of RAR target genes. Accordingly, we found that expression of genes encoding RA degradation enzymes, cytochrome P450 26a1 (Cyp26a1; eight-fold induction) and Cyp26b1 (two-fold increase) was increased in allergen-induced dermatitis (Table 3). Expression of proteins involved in retinoid transport (Rbp1, Crabp2) and metabolism (Lrat) was similarly increased in the skin of mice treated with OVA, regardless of further topical sensitization with OVA, whereas expression of RAR target genes not involved in retinoid signaling (Krt4, Rarres2, Tgm2) was not significantly altered (Table 3). Notably, the ratio of Fabp5 vs. Crabp2 gene expression, both delivering ATRA to their respective cognate receptors, significantly increased in allergen-induced dermatitis (Figure 2c). Thus, allergen-induced dermatitis might lead to increased ATRA levels in the skin and dysregulated retinoid metabolism and signaling at least in our mouse model of the disease.

**Table 3:** Systemic and topical OVA sensitizations induce retinoic acid synthesis and dysregulate retinoid-mediated signaling in skin of mice.

		Fold change	
Gene name	Symbol	OVA i.p.	OVA i.p.+e.c.
**Retinal synthesis**			
Short chain dehydrogenase/reductase 16C5	Sdr16c5	1.7±0.2*	1.8±0.2*
Retinol dehydrogenase 10	Rdh10	1.1±0.1	1.3±0.1
**Retinoic acid synthesis**			
Aldehyde dehydrogenase 1A1	Aldh1a1	1.8±0.2	2.4±0.4**
Aldehyde dehydrogenase 1A2	Aldh1a2	0.5±0	3.9±1.3* #
Aldehyde dehydrogenase 1A3^1^	Aldh1a3	4.8±0.4**	4.0±0.8**
**Retinoid receptors**			
Retinoic acid receptor α	Rara	0.8±0.1	1.0±0.1
Retinoic acid receptor β^1^	Rarb	0.8±0.1	0.9±0.1
Retinoic acid receptor γ	Rarg	0.8±0.1	1.3±0.2#
Retinoid X receptor α	Rxra	0.7±0.1	1.6±0.2* ^###^
**RAR target genes involved in retinoid signaling**			
*Retinoic acid degradation*			
Cytochrome P450 26A1	Cyp26a1	2.1±0.7	7.9±2.2** #
Cytochrome P450 26B1	Cyp26b1	0.6±0.1	1.9±0.2* ##
*Retinoid transport proteins*			
Cellular retinol binding protein 1	Rbp1	3.5±0.2***	3.0±0.2***
Cellular retinoic acid binding protein 2	Crabp2	1.3±0.1	1.4±0.1
*Retinol esterification*			
Lecithin-retinol acyltransferase	Lrat	2.4±0.3	2.5±0.7
*Further RAR target genes not involved in retinoid signaling*			
Keratin 4	Krt4	0.6±0.2	0.3±0*
Retinoic acid receptor responder 2	Rarres2	0.5±0.1*	0.6±0.1
Transglutaminase 2	Tgm2	0.9±0.1	0.7±0.1

e.c., epicutaneous; i.p., intraperitoneal; OVA, ovalbumin.

1 RAR target genes.

Fold change data are expressed as mean ± SEM (n = 6) and were determined in skin specimen of sensitized mice by TLDA. Statistical significance (*p*) was tested using one-way ANOVA followed by Tukey’s multiple comparison test.

* *p*<0.05,

** *p*<0.01,

*** *p*<0.001, versus control (PBS i.p.);

# *p*<0.05,

## *p*<0.01, and ^###^ *p*<0.001, versus OVA i.p.

### Gene Targets Involved in and Mediated by PPARδ Pathways in Skin are Mainly Up-regulated in Allergen-induced Dermatitis

Gene expression of PPARδ as well as several of its target genes in skin is presented in Table 2. Systemic or systemic plus topical sensitization of mice with OVA led to reduced PPARδ gene expression compared to controls and this decrease was somewhat more pronounced in mice systemically sensitized only. In contrast, mRNA expression of Fabp5, the fatty acid binding protein which delivers ligands to PPARδ, was increased after sensitization with OVA (Table 2). Moreover, keratin 6b (Krt6b), keratin 16 (Krt16), heparin-binding EGF-like growth factor (Hbegf) and Hmgcs2, all of which known to be induced upon PPARδ activation and involved in epidermal barrier homeostasis [18], [32], [33], showed significantly elevated gene expression levels in skin after systemic and topical sensitization. Only the PPARδ target gene Abca12 [34], which is responsible for epidermal barrier formation and maintenance, showed decreased mRNA levels in both OVA treatment groups (Table 2). Altogether, our results suggest an induction of gene targets which are involved in PPARδ signaling pathways, most noticeably Fabp5, in murine skin in response to systemic and topical OVA sensitization.

### Systemic Sensitization with OVA Increases Fabp5 Protein Levels

**Figure 3:**
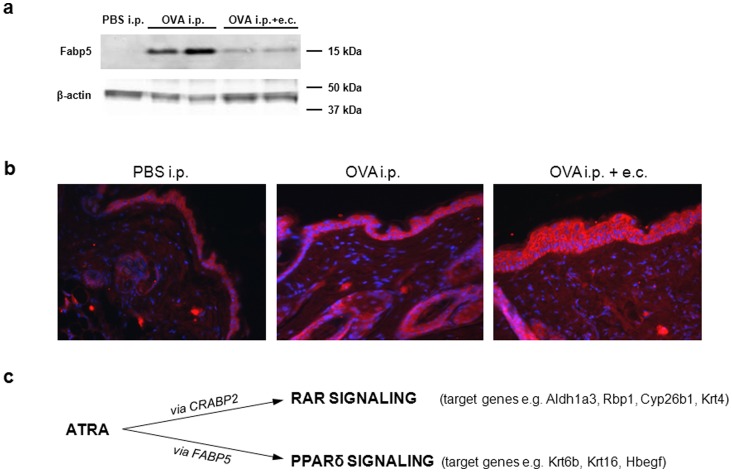
Increased Fabp5 expression in allergen-induced dermatitis. (**a**) Fabp5 protein levels in the skin of mice with allergen-induced dermatitis. 150 µg proteins were loaded per lane and beta-actin was used as control for even protein loading. (**b**) Immunohistochemical analysis of Fabp5 protein expression in five-micrometer back skin sections of OVA-sensitized mice. (**c**) ATRA-induced nuclear receptor-mediated signaling pathways depending on the predominant cellular transport protein.

Because Fabp5 gene expression in skin was induced after repeated systemic OVA sensitization (Table 2), we next assessed levels of Fabp5 protein in the skin of mice in our various experimental conditions. Levels of Fabp5 protein as measured by Western Blots, increased in skin of mice systemically sensitized with OVA compared to controls (Figure 3a). However, highest Fabp5 protein levels were detected in whole skin of mice systemically treated with OVA (Figure 3a). In order to determine the localization of Fabp5 across the skin, we performed immunohistochemical analysis. We found intense staining for Fabp5 in the thickened epidermis and around hair follicles of mice treated with OVA (Figure 3b). Thus, systemic sensitization with OVA is sufficient to increase levels of Fabp5 in the skin of mice.

## Discussion

The present study demonstrates that the immune response in allergen-induced dermatitis is associated with increased retinoid signaling and RA concentrations in the skin. Moreover, signaling via PPARδ-mediated pathways, mostly through Fabp5 upregulation, was mainly enhanced in allergen-induced dermatitis. Thus, retinoid-mediated signaling is involved in the pathogenesis and/or maintenance of allergic dermatitis or further atopic skin diseases such as AD, but the exact pathway is not yet determined.

High RA levels in the skin, as observed in the present work, might directly impact on systemic and local immune responses [14], [35]–[38]. In our mouse model of allergen-induced dermatitis, we found a mixed Th1- and Th2-type immune response in the skin and high numbers of infiltrating dermal macrophages, dendritic cells and mast cells (Table 1 and 2). In contrast, mice systemically treated with OVA exhibited only a partial phenotype with lower inflammatory infiltrates and cytokine expression in the skin. Interestingly, the highest levels of immune response-related gene expression, inflammatory cell infiltrates and serum cytokines correlated with increased expression of RA synthesizing enzymes and ATRA levels in inflamed skin. Thus, these data suggest that only overt allergen-induced dermatitis leads to an increased ATRA concentration and altered RA signaling in the skin.

The increased ATRA levels in the skin of OVA-sensitized mice (Figure 2b, Table S1) might reflect the induced expression of RA synthesizing enzymes (Table 3) that in turn may result in elevated ATRA synthesis in murine skin. However, besides resident skin cells, infiltrating immune cells might be a source of ATRA in sensitized skin. For example, human basophils which have been shown to infiltrate AD skin [39] were found to express Aldh1a2 enzyme and to produce RA upon activation with IL-3 in an *ex vivo* model [40]. However, identification of specific cell types producing RA in inflamed skin is currently not feasible due to problems in acquiring sufficiently large numbers of highly purified cells from the skin.

One of the major outcomes of the present work was to demonstrate that systemic sensitization of mice per se is sufficient to induce partial skin immune responses and an impairment of expression of key genes involved in skin homeostasis and barrier function (Table 1 and 2, Figure 2a). Previous studies and reviews reported an “outside-inside-outside” pathogenic mechanism of AD [4]–[6]. In contrast, our data support an “inside-out” mechanism significantly contributing to the development of overt skin inflammation.

It has previously been shown that ATRA is not only ligand of RARs but can also activate PPARδ and induce PPARδ target gene expression. PPARδ signaling is favored instead of RAR pathways when the ratio of the lipid transporters Fabp5 vs. Crabp2 is high within cells such as keratinocytes [19], [20]. We determined highest Fabp5 protein levels in the skin of mice treated systemically with OVA (Figure 3a). In contrast, immunohistochemical analysis showed particularly intense staining in the epidermis and around hair follicles of mice with allergen-induced dermatitis (Figure 3b). In the literature, Fabp5 protein is described to be predominantly present in epidermis [41], sebaceous glands and hair follicles [42] and in subcutaneous adipocytes [43]. However, in our study western blot analysis was performed from whole skin, therefore, a larger increase of Fabp5 protein expression in dermis and/or subcutaneous fat after systemic OVA treatment compared to systemic and topical treatment may explain the apparent discrepancy between Figures 3a and 3b. Notably, in our mouse model, the Fabp5 vs. Crabp2 ratio was increased in allergen-induced dermatitis (Figure 2c). This data might suggest favored ATRA signaling through PPARδ which may significantly contribute to the specific gene expression patterns observed in this study (see below and indicated in Figure 3c). PPARδ signaling and several of its target genes were previously found increased in psoriasis and lesional AD skin [18], [33], [44] and Romanowska et al. [18] further demonstrated the induction of an inflammatory skin disease similar to human psoriasis in PPARδ-overexpressing mice. Interestingly, in our mouse model of allergen-induced dermatitis we observed an increased expression of several of the investigated target genes involved in PPARδ signaling pathways in skin. Though further investigations potentially involving PPARδ knockout mice would be required to confirm these data, our results suggest favored ATRA-mediated PPARδ signaling in allergen-induced dermatitis.

Most notably, several RAR target genes involved in retinoid signaling were induced in allergen-induced dermatitis, whereas expression of RAR targets which are not implicated in retinoid signaling (Krt4, Rarres2, Tgm2) was not significantly altered. Thus, expression of genes involved in RA synthesis as well as degradation, transport and esterification, and especially of RAR target genes was increased in allergen-induced dermatitis. In contrast, expression of RAR target genes rather related to epidermal differentiation remained unaltered or reduced. These data thus indicate that potentially increased ATRA synthesis via Aldh1a enzymes and elevated ATRA levels in mouse skin observed in allergen-induced dermatitis might not result in an overall increase of RAR-mediated signaling. Furthermore, OVA treatment might also affect the level of several other lipids and nuclear receptor agonists than ATRA in mouse skin.

In summary, allergen-induced dermatitis is associated with increased retinoid signaling and elevated ATRA levels in the skin. Because expression of genes involved in all aspects of RA metabolism is increased, whereas expression of RAR target genes involved in other pathways such as epidermal differentiation remains largely unchanged, allergen-induced dermatitis might additionally redirect intracellular retinoid flux and metabolism. Moreover, PPARδ gene targets were mainly induced indicating that RAR-mediated signaling and certain pathways/molecules involved in PPARδ signaling are altered in allergic dermatitis skin. Furthermore, systemic sensitization with an allergen is sufficient to modify the expression of genes central to epidermal homeostasis suggesting an “inside-out” effect of allergen in allergic skin disease pathogenesis possibly by increasing allergen penetration through the skin. Whether disturbed retinoid metabolism and retinoid-mediated signaling are symptoms or potential initiators of atopic sensitization still remains to be elucidated.

## Supporting Information

Table S1 **Systemic and topical OVA sensitizations result in increased all-** ***trans*** **retinoic acid levels in skin.**
(DOC)

Materials and Methods S1 **Immunohistochemical analysis.**
(DOC)

Materials and Methods S2 **Determination of FABP5 protein in skin.**
(DOC)

Materials and Methods S3 **Protocol for the determination of all-** ***trans*** **retinoic acid levels in skin by HPLC MS-MS method.**
(DOC)
