# Supplementary material for: Allergen-Induced Dermatitis Causes Alterations in Cutaneous Retinoid-Mediated Signaling in Mice
Source: PLoS One. 2013 Aug 15;8(8):e71244. doi: 10.1371/journal.pone.0071244 (PMC3744553; doi:10.1371/journal.pone.0071244)
Supplement: Table S1 — Systemic and topical OVA sensitizations result in increased all- trans retinoic acid levels in skin. (DOC) [file pone.0071244.s001.doc]

**Table S1. Systemic and topical OVA sensitizations result in increased all-*trans* retinoic acid levels in skin.**

| **Retinoid concentrations in ng/g** | **PBS i.p.** | **OVA i.p.** | **OVA i.p.+e.c.** |
| --- | --- | --- | --- |
| all-*trans* retinoic acid (ATRA) | 0.5 ± 0.2 | 0.8 ± 0.2 | 1.4 ± 0.2^*^ |
| retinol (ROL) | 100 ± 14 | 126 ± 36 | 136 ± 13 |

e.c., epicutaneous; i.p., intraperitoneal; OVA, ovalbumin; PBS, phosphate-buffered saline.

Concentrations are expressed as ng/mg with mean ± SEM calculated (n=3) and were determined by HPLC MS-MS method in skin specimen of control mice and OVA-sensitized mice. Statistical significance (*p*) was tested using student’s *t*-test. **p*<0.05 vs. PBS i.p.
