# Supplementary material for: Allergen-Induced Dermatitis Causes Alterations in Cutaneous Retinoid-Mediated Signaling in Mice
Source: PLoS One. 2013 Aug 15;8(8):e71244. doi: 10.1371/journal.pone.0071244 (PMC3744553; doi:10.1371/journal.pone.0071244)
Supplement: Materials and Methods S1 — Immunohistochemical analysis. (DOC) [file pone.0071244.s002.doc]

**Supporting Materials and Methods S1. Immunohistochemical analysis**

**(a)** Frozen five-micrometer skin sections were fixed in acetone, blocked with mouse seroblock FcR block (AbD Serotec, Düsseldorf, D) or 10% goat serum (NGS; Vector Laboratories) and incubated with FITC rat anti-mouse CD3 molecular complex (17A2); biotin rat anti-mouse CD8a (53-6.7); purified hamster anti-mouse CD11c (HL3; all BD Biosciences - Pharmingen, San Diego, CA); or purified rat anti-mouse CD4 (GK1.5; BioLegend, San Diego, CA). Antibody binding was detected using biotinylated goat anti-rat Ig for anti-CD4 (Amersham Biosciences UK limited) and biotin mouse anti-hamster IgG cocktail for anti-CD11c (BD Biosciences - Pharmingen, San Diego, CA), followed by incubation with Alexa Fluor 594-linked streptavidin (Invitrogen, Carlsbad, CA) for anti-CD4, anti-CD11c and anti-CD8. Skin sections stained for CD11c were counterstained with FITC-linked rat anti-mouse I-A/I-E (BD Biosciences - Pharmingen, San Diego, CA) to identify MHC class II-positive cells. Nuclei were visualized with DAPI.

**(b)** IHC staining of paraffin-embedded skin sections with rabbit Fabp5 polyclonal antibody (1:50; ProteinTech, Chicago, IL) was performed following the manufacturer’s directions using antigen retrieval buffer (0.1 M sodium citrate, 0.1 M citric acid) and blocking with 5% donkey serum. Biotinylated donkey anti-rabbit Ig (Amersham Biosciences UK limited) and Alexa Fluor 594-linked Streptavidin were applied for detection of antibody binding.

All sections were mounted with Vectashield Mounting Medium (Vector Laboratories, Burlingame, CA).
