# Supplementary material for: Allergen-Induced Dermatitis Causes Alterations in Cutaneous Retinoid-Mediated Signaling in Mice
Source: PLoS One. 2013 Aug 15;8(8):e71244. doi: 10.1371/journal.pone.0071244 (PMC3744553; doi:10.1371/journal.pone.0071244)
Supplement: Materials and Methods S2 — Determination of FABP5 protein in skin. (DOC) [file pone.0071244.s003.doc]

**Supporting Materials and Methods S2. Determination of FABP5 protein in skin**

Skin was lysed in RIPA lysis buffer in the presence of protease inhibitors (Pierce, Rockford, IL). Lysates were separated by 4-12% SDS-PAGE and then transferred to a nitrocellulose membrane (Invitrogen, Carlsbad, CA). The membrane was incubated at room temperature in a blocking solution (Pierce, Rockford, IL) for 30-60 min. The membrane was then incubated with the FABP5 antibody (1:500, ProteinTech, Chicago, IL) diluted in blocking buffer overnight at 4°C. Endogenous proteins were detected with Alexa A680-conjugated anti-rabbit secondary antibody (1:10.000 dilution) (Invitrogen, Carlsbad, CA). Blots were then scanned with a LI-COR Biosciences analyzer. Anti-beta-actin from Sigma (St Louis, MO) was used as loading control.
