# Supplementary material for: Allergen-Induced Dermatitis Causes Alterations in Cutaneous Retinoid-Mediated Signaling in Mice
Source: PLoS One. 2013 Aug 15;8(8):e71244. doi: 10.1371/journal.pone.0071244 (PMC3744553; doi:10.1371/journal.pone.0071244)
Supplement: Materials and Methods S3 — Protocol for the determination of all- trans retinoic acid levels in skin by HPLC MS-MS method. (DOC) [file pone.0071244.s004.doc]

**Supporting Materials and Methods S3. Protocol for the determination of all-*trans* retinoic acid levels in skin by HPLC MS-MS method**

In summary, 100 mg of skin biopsy (if samples were under 100 mg, water was added up to the used standard weight: 100 mg) were diluted with a threefold volume of isopropanol, tissues were minced by scissors, vortexed for 10 seconds, put in an ultra sonic bath for 5 minutes, shaken for 6 minutes and centrifuged at 13000 rpm in a Heraeus BIOFUGE Fresco at +4 °C. After centrifugation, the supernatants were dried in an Eppendorf concentrator 5301 (Eppendorf, Germany) at 30 °C. The dried extracts were resuspended with 60 μl of methanol, vortexed, shaken, diluted with 40 μl of 60 mM aqueous ammonium acetate solution and transferred into the autosampler for subsequent analysis.
